# Brace treatment can serve as a time-buying tactic for patients with congenital scoliosis

**DOI:** 10.1186/s13018-019-1244-4

**Published:** 2019-06-27

**Authors:** Yuwen Wang, Zongxian Feng, Zhichong Wu, Yong Qiu, Zezhang Zhu, Leilei Xu

**Affiliations:** 1 The Affiliated Drum Tower Hospital of Nanjing University Medical School, Zhongshan Road 321, Nanjing, 210008 China; 2 Ningbo Medical Center Lihuili Eastern Hospital, No. 57 Xingning Road, Ningbo, 315000 China

**Keywords:** Congenital scoliosis, Brace treatment, Effectiveness

## Abstract

**Background:**

Infantile patients with congenital scoliosis (CS) can be confronted with increasing risk of mortality and morbidity. To date, the effectiveness of conservative treatment in CS has not been sufficiently investigated. We aimed to evaluate the bracing outcome in patients with CS and to investigate whether wearing brace can effectively delay the surgical procedures.

**Methods:**

A total of 39 braced CS patients including 25 boys and 14 girls were reviewed for the eligibility to be included in this study. Radiographic parameters including curve magnitude and T1 to T12 height were evaluated for each patient at the initiation of the treatment and at the final follow-up (FU), respectively. Duration of the follow-up and requirement of surgical interventions were also recorded. The student *t* test was used to compare the radiographic parameters between the initial visit and the last FU.

**Results:**

The mean initial age at bracing was 4.1 ± 2.3 years, and 7.5 ± 1.8 brace modifications were performed during a mean FU period of 42.1 ± 26.5 months. The mean curve magnitude before bracing was 44.1 ± 12.2°, which was corrected to 41.3 ± 13.5° at the final visit (*p* = 0.33). T1-T12 height increased from 13.4 ± 2.5 to 17.1 ± 2.8 cm during the treatment (*P* < 0.001). Nine patients underwent surgical intervention due to the curve progression more than 5°, with the time of surgery delayed for 32.1 ± 18.2 months.

**Conclusions:**

Brace treatment is an effective time-buying modality for CS patients, which may help maintain the body growth and delay the surgical intervention.

## Background

Congenital scoliosis (CS) is a rare type of spinal deformity secondary to congenital vertebral malformation (CVM) with an incidence of 0.1% approximately [1]. Usually, the severity of the congenital scoliosis is dependent on CVM type, location and number, as well as the patients’ age [2–4]. Previous studies have demonstrated that most congenital scoliosis may be progressive [5–7]. McMaster et al. [8] concluded that curve progression depends on the type of CVM and the spinal region involved. Hemivertebra and thoracic curves are associated with the poorest prognosis. Without appropriate treatment, infantile patients with CS can be confronted with increasing risk of mortality and morbidity concomitant with cardiopulmonary dysfunction [9].

Progressive CS in the immature spine poses unique management challenges for the surgeons with limited intervention modalities available. Several treatment options exist to correct deformity or prevent progression, including casting, in situ fusion, growing rods, and vertical expandable prosthetic titanium rib [10–13]. In earlier literatures concerning treatment of patients with CS, the choice of age at surgery was still in debate [14–16]. Some authors proposed to perform surgery such as epiphysiodesis or CVM resection at a very early age, while others argued that fusion surgery at early stage of the patients was not an effective means of treatment due to the premature growth arrest of the spine and thoracic cage [15, 16]. Moreover, multiple surgical exposures and an increased risk of associated complications such as infections and implant failure have confined the effectiveness of early-stage surgery for CS. Gradually, it was well accepted that desirable correction treatment should allow for continued spinal growth and maturation of the lung tissues which commonly occurs at the age of 8 years [15]. Thus, before this age, delaying tactics alternative to fusion should be actively applied to the patients.

Cast correction represents another alternative for scoliosis, which was widely used before spinal instrumentation [17–19]. Recent studies showed that serial casting may help delay the growing rod surgery for early-onset scoliosis (EOS), which could decrease the incidence of complications related to surgical procedures [19–21]. Conservative techniques are conventionally considered inappropriate for patients with CS due to the progressive spinal deformity. Nevertheless, taking advantage of delay of the initial surgery, recently serial casting has been applied to the treatment of CS [10, 11]. Demirkiran et al. [11] reviewed a total of 11 CS patients treated with serial cast application and reported that it is a safe and effective time-buying strategy to delay the surgical interventions in congenital deformities. Cao et al. [10] also reported that casting is an efficient treatment option to delay the surgery for CS patients. As the aforementioned reports included a small cohort of patients, the role of serial casting in CS has not been sufficiently investigated. As a conservative modality similar with serial casting, brace treatment has been applied to CS patients of our clinic center for a few years. The objectives of this study were to evaluate the bracing outcome in patients with CS, and to investigate whether wearing brace can effectively delay the surgical procedures.

## Methods

### Subjects

Under the approval of the local Institutional Review Board, we reviewed all the patients undergoing brace treatment for EOS between May 2005 and June 2015 at our scoliosis center. The inclusion criteria were as follows: (1) diagnosed as congenital scoliosis or infantile idiopathic scoliosis (IIS), (2) with an initial Cobb angle of less than 50°, (3) aged younger than 8 years, (4) receiving no other treatment prior to bracing, and (5) with the bracing period longer than 12 months. The exclusion criteria were as follows: (1) with short and sharp angular congenital deformities secondary to hemivertebra and (2) with the treatment discontinued due to complications such as neurological impairment and skin irritation. A cohort of 39 CS patients and 24 IIS patients were finally included in the study.

### Bracing strategy

For CS patients, apical region of the kyphosis was well padded to prevent skin ulcers. Each patient was initially instructed to wear the brace for 22 h. Routine follow-up visit was carried out at an interval of 3 month, and the brace was modified according to the growth status of the patients. The brace treatment was discontinued in case of curve progression beyond 50°. Standard standing posteroanterior and lateral out-of-cast radiographs were taken at each visit for radiographic evaluation.

### Data collection

Baseline demographics including initial age, Risser sign, curve pattern, duration of treatment, number of brace modified, requirement of surgery, and bracing-related complications were collected. Radiographic measurements including major curve magnitude, T1 to T12 height, coronal balance, and sagittal balance were evaluated for each patient at the initiation of the treatment and at the final visit, respectively. The coronal balance was evaluated by the horizontal distance between C7 plumb line (C7PL) and center sacral vertical line (CSVL). The sagittal balance was evaluated by sagittal vertical axis (SVA).

### Statistical analysis

The software Statistical Package for the Social Sciences (SPSS, version 19.0) was used for statistical analyses. Continuous variables were summarized as the mean value ± SD. The correction rate was calculated as follows: (pre-brace angle − final-visit angle)/pre-brace angle × 100%. The Student *t* test was used for comparisons of continuous variables. The Chi-square test was used for comparisons of categorical variables. Statistical significance was set at a *p* value of 0.05.

## Results

The baseline characteristics of the subjects were summarized in the Table 1. The two groups were matched in terms of initial age, gender, and initial curve magnitude. For the CS group, there were 14 female and 25 male patients. All patients had long congenital curves with formation (*n* = 27) or segmentation anomalies (*n* = 12). Thirty-three patients had main thoracic curve and six patients had lumbar or thoracolumbar curve. As for skeletal maturity, all the patients had an initial Risser sign of stage 0. The mean initial age was 4.1 ± 2.3 years (range, 1.5 to 7 years). The average number of brace modifications was 7.5 ± 1.8 (range, 2 to 12 times). The mean period of brace treatment was 42.1 ± 26.5 months (14–118 months).

**Table 1:** The baseline characteristics of the CS patients and the IIS patients

	CS (*n* = 39)	IIS (*n* = 24)	*P*
Age (years)	4.1 ± 2.3	4.3 ± 2.1	0.73
Ratio of male to female	25: 14	15: 9	0.88
Duration of brace treatment (months)	42.1 ± 26.5	48.4 ± 13.7	0.19
Number of brace modifications	7.5 ± 1.8	6.8 ± 1.5	0.08
Initial curve magnitude (°)	44.1 ± 12.2	38.5 ± 13.5	0.10

**Fig. 1:**
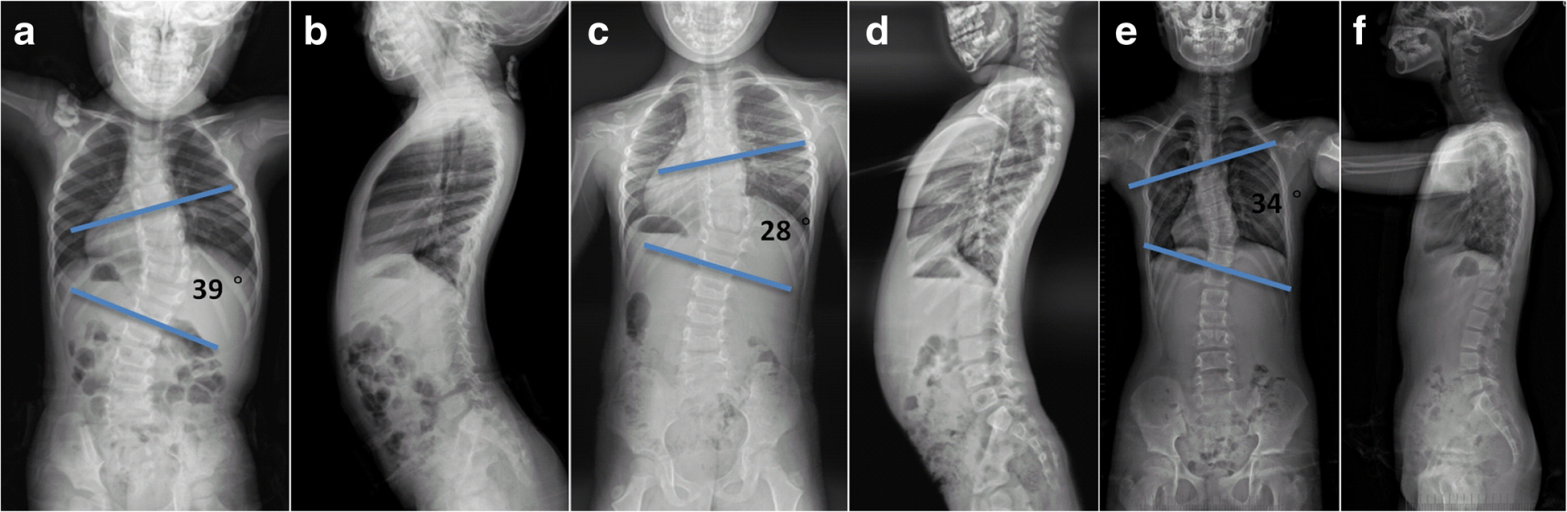
Radiographs of a patient with favorable outcome of bracing. **a**, **b** A 2-year-old female CS patient. Before brace treatment, the curve magnitude was 39°. The T1-T12 height was 129.9 mm. The coronal balance and the sagittal balance were 12.8 mm and 28.34 mm, respectively. **c**, **d** 28 months after brace treatment, the curve magnitude was corrected to 28°. The T1-T12 height increased to 157.8 mm. The coronal balance and the sagittal balance were 12.5 mm and 3.8 mm, respectively. **e**, **f** 54 months after brace treatment, the curve magnitude was 34°. The T1-T12 height increased to 175.6 mm. The coronal balance and the sagittal balance were 9.8 mm and 11.9 mm, respectively

**Fig. 2:**
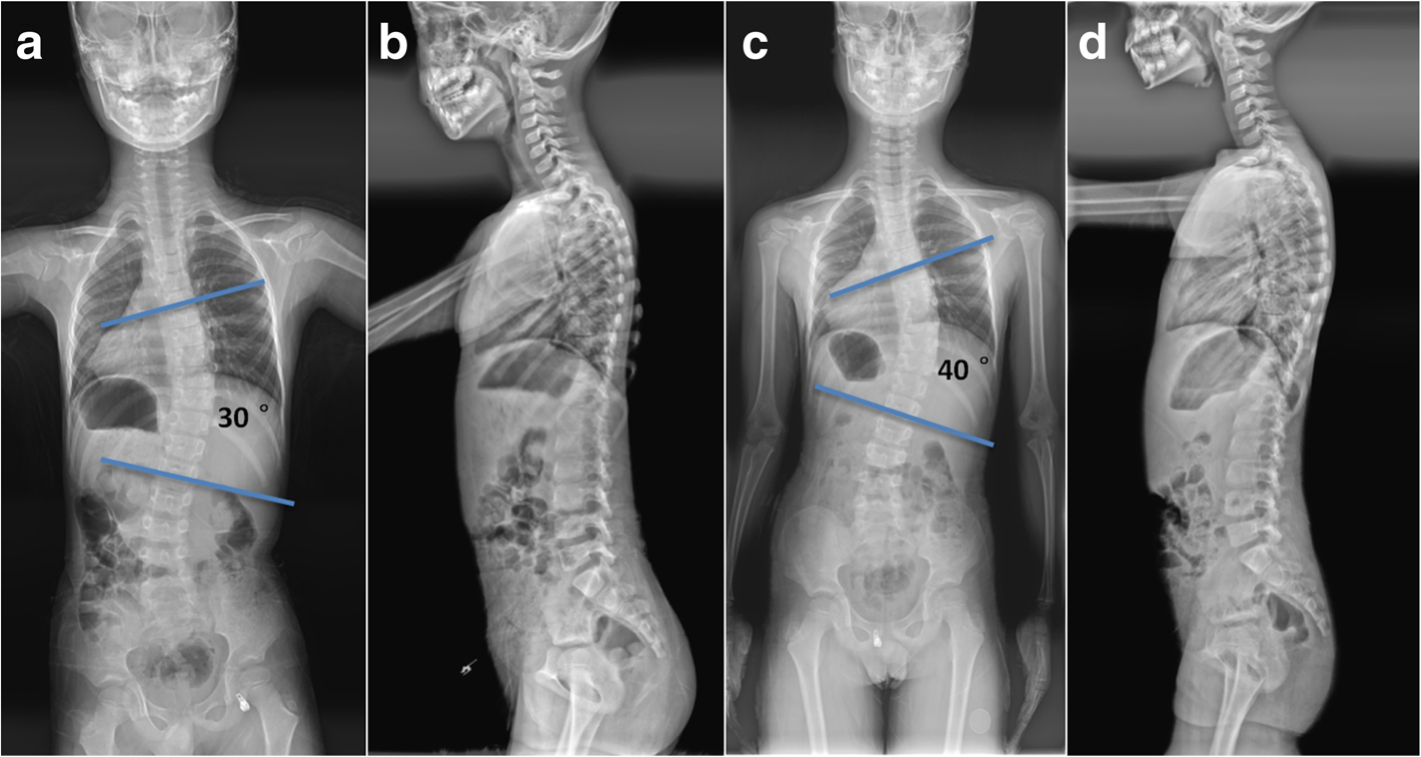
Radiographs of a patient with curve progression after bracing. **a**, **b** A 6-year-old male CS patient. Before brace treatment, the curve magnitude was 30°. The T1-T12 height was 176.5 mm. The coronal balance and the sagittal balance were 12.7 mm and 11.4 mm, respectively. **c**, **d** 19 months after brace treatment, the curve magnitude progressed to 40°. The T1-T12 height increased to 193.4 mm. The coronal balance and the sagittal balance were 10.1 mm and 15.2 mm, respectively

The radiographic data of the subjects were summarized in the Table 2. For CS patients, the mean curve magnitude before bracing was 44.1 ± 12.2°, which was corrected to 41.3 ± 13.5° at the final visit (*p* = 0.33). Twenty (51.3%) patients were found to have curve correction of more than 5° (Fig. 1). Nine (23.1%) patients were found to have curve progression of more than 5° (Fig. 2). Kyphotic deformity was observed in eight patients. The local kyphosis angle was reduced from 58.4 ± 11.3° to 52.3 ± 11.9° (*p* = 0.02). The mean T1 to T12 height before the brace treatment was 13.4 ± 2.5 cm. At the final visit, it significantly increased to a mean value of 17.1 ± 2.8 cm (*p* < 0.001), with an average growth rate of 1.02 ± 0.21 cm/year. Before bracing, the coronal and sagittal balance were averaged 13.4 ± 9.1 mm and 23.6 ± 13.9 mm, which were reduced to 12.1 ± 6.2 mm and 18.5 ± 13.4 mm at the latest follow-up (*p* = 0.46 for C7PL-CSVL; *p* = 0.10 for SVA), respectively. As shown in Table 2, patients with IIS were found to have significantly better correction rate than patients with CS (28.2% ± 11.6% vs. 14.8% ± 13.5%, *p* < 0.001). The incidence of curve progression was higher in the CS group than in the IIS group (23.1% vs. 16.7%, *p* = 0.54). The growth rate of thoracic spine was comparable between the two groups (1.02 ± 0.21 vs. 1.07 ± 0.18, *p* = 0.33).

**Table 2:** Comparison of bracing outcome in the two groups

	CS (*n* = 39)	IIS (*n* = 24)	*P*
Final curve magnitude (°)	41.3 ± 13.5	29.8 ± 10.4	< 0.001
Curve correction rate	14.8% ± 13.5%	28.2% ± 11.6%	< 0.001
Incidence of curve progression	23.1%	16.7%	0.54
Growth rate of T1-T12 (cm/year)	1.02 ± 0.21	1.07 ± 0.18	0.33

At the time of the last visit, growing rod surgery was performed for nine patients, who had worn the brace for an average of 32.1 ± 18.2 months (range, 14–59 months). The mean age at surgery was 5.6 ± 2.2 years (range, 4–10 years). As shown in Table 3, there was no significant difference between patients with curve progression and those without curve progression in terms of initial age, curve magnitude, or curve pattern. As for the complications, five patients in the CS group reported skin ulcers due to the kyphotic deformity, which were subsequently alleviated after the modification of the brace. There was no other type of complication for both groups.

**Table 3:** Assessment of variables related to curve progression in CS patients

Variables	Patients with curve progression (*n* = 9)	Patients without curve progression (*n* = 30)	*P*
Initial age	3.6 ± 1.6	4.3 ± 2.1	0.37
Initial curve magnitude	47.1 ± 11.5	43.2 ± 12.6	0.41
Curve pattern			0.63
Thoracic curve	8	25	
Thoracolumbar/lumbar curve	2	4	

## Discussion

Treatment of CS in infantile and juvenile patients remains a great challenge in clinical practice. Growth-friendly techniques such as growing rods and vertical expandable prosthetic titanium rib (VEPTR) gained popularity in management of all types of EOS, including congenital scoliosis [12, 13, 15]. However, it is still under debate whether surgical intervention can alter the natural history of CS with a favorable outcome. Both above-mentioned methods require frequently recurrent surgical interventions and have a high incidence of complications. On the other side, serial casting or bracing as a standalone treatment method for scoliosis in young children has been widely used. Most current studies supported their role as a definitive treatment modality in the management of mild curves for patients with EOS [15, 18, 21]. As for the CS, however, few data are available since most congenital scoliotic curves are believed to be resistant to serial casting and bracing.

To investigate whether bracing can be effectively used for the treatment of CS, we reviewed the bracing outcome in a cohort of 39 CS patients. In this study, significant correction of the major curve was observed at the latest visit, with a mean correction rate of 14.8%. Over half of the patients (51.3%) were observed to have an improvement of the curve that was more than 5°. Comparably, Demirkiran et al. [11] have reported a correction rate of 22% in 11 CS patients receiving casting treatment. Ten out of the 11 patients had the curve corrected by more than 5°. In the study of Cao et al. [10], the mean correction rate was 20.5% for the CS group. Taken together, the curve progression in CS patients can be controlled through bracing, and the therapeutic effects were maintained well. Moreover, we compared variables between patients with curve progression and those without curve progression, while no significant difference in terms of these variables was observed. It was probably due to the small sample size that leads to weak statistical power to detect influential factors. In future study, recruitment of more CS patients who received brace treatment is warranted to investigate factors associated with curve progression.

In previous studies, serial casting has been proven an effective treatment to delay the first surgery for EOS patients [18, 21]. Fletcher et al. [21] reported 39 months of delay in surgery for 17 EOS patients, and 72.4% of them were saved from growing rod surgery. Likewise, Demirkiran et al. [11] reported an average of 26.3 months of delayed surgery in 11 CS patients receiving serial casting. Cao et al. [10] reported that casting treatment delayed the time of first surgery by an average of 15 months for two CS patients. To be noted, these recent reports on CS included a small number of patients with congenital deformities. In this study, nine of the 39 CS patients finally underwent correction surgery after a mean bracing period of 32.1 months. For the other 30 patients, the surgery had been successfully avoided for a mean period of 45.9 months. Apparently, bracing had effectively delayed the time of first surgery for CS patients, although most of them may eventually need surgical interventions.

An important goal of recurrent surgery in children with CS is to maintain the spinal growth and the development of pulmonary function. Under the traction force in casting correction, serial casting correction would help spinal growth according to Hueter-Volkmann law. To investigate how much of this goal could be achieved through brace treatment, we measured spinal height from the T1 to T12 for each patient. Remarkable spinal growth was observed with an average growth rate of 1.02 cm/year. This finding is similar to a mean thoracic growth rate of 0.72 cm/year reported by Cao et al. and 0.81 cm/year reported by Demirkiran et al. [10, 11]. It is noteworthy that the spinal grow rate of braced CS patients was lower than the normal range of age-matched children (1.5 cm/year) [22]. However, considering the congenital deformities of the patients, we believed that bracing could effectively preserve the growth potential of the spine.

In this study, a comparison between the CS group and the IIS group showed that brace treatment could benefit both groups of patients, while the therapeutic outcome of CS patients was less favorable than that of the IIS patients. Compared with IIS patients, CS patients have a relative rigid spine, which is more difficult to be corrected by the traction and derotational force of brace treatment. Obviously, bracing can only serve as a delaying tactic but not a definitive treatment method for young patients with CS.

One limitation of the present study needs to be addressed. As an inherent drawback of retrospective study, the small sample size and short follow-up duration may compromise the power of statistical analysis. A multi-center prospective study with larger sample size and long-term follow-up is warranted for further assessment of the therapeutic effect of bracing.

## Conclusions

The primary purpose of conservative treatment in CS patient is to delay the surgical interventions and decrease the number of recurrent surgical procedures. We confirmed that bracing is a safe and effective time-buying strategy to delay the time of surgical interventions for congenital scoliosis. The curve progression can be well controlled through bracing, and the growth potential of the spine can be effectively preserved.

## Abbreviations

C7PL: C7 plumb line
CS: Congenital scoliosis
CSVL: Center sacral vertical line
CVM: Congenital vertebral malformation
EOS: Early-onset scoliosis
IIS: Infantile idiopathic scoliosis
SPSS: Software Statistical Package for the Social Sciences
SVA: Sagittal vertical axis
VEPTR: Vertical expandable prosthetic titanium rib
